# Abstracts of paper presented at the 2000 Pittsburgh Conference

**DOI:** 10.1155/S1463924601000049

**Published:** 2001


Journal of Automated Methods & Management in Chemistry, Vol. 23, No. 2 (March–April 2001) pp. 41–58

### Abstracts of paper presented at the 2000 Pittsburgh Conference

The Pittsburgh Conference was held from 12–17 March 2000 in New Orleans, USA. The papers and posters were up to the usual high standard and as in the past a number of abstracts were selected for publication in this journal. However, due to a technical oversight on the Editor's behalf (for which I apologize) these abstracts were not scheduled for production.

I have therefore selected a smaller number for presentation here. If your abstract has been omitted then please accept our apologies, the abstracts presented here would appear to have the most useful proposals and comments relevant to our publication.

We look forward to the next Pittsburgh Conference in March 2001 which will also be held in New Orleans.

Peter B. Stockwell
*Editor*

### Micro-devices fabricated in plastics using lithography and injection moulding for capillary electrophoretic applications

*Shize Qi, Sean Ford and Steven Soper, Louisiana State University, Department of Chemistry, Baton Rouge, LA 70803*

X-ray Lithography was used to transfer the design of the microfabricated device to a plating base. A graphite sheet was spin-coated with a negative photoresist. The design was printed onto a graphic sheet by UV exposure. After removing the exposed photoresist, gold was electroplated on the graphite to form an X-ray mask, which was used to expose a PMMA sheet (120 µm) on a plating base using X-rays. Nickel was then electroplated onto the plating base to generate an insert for injection moulding. Using this technique, we could fabricate a device with extremely smooth walls and high aspect ratios. In addition, a variety of different polymers can be moulded, such as PMMA. Microfabrication devices can be obtained after demoulding the PMMA and thermally bonding (using low temperatures) the cover plate (PMMA) to the device.

Because we could produce deep and narrow channels, fused silica capillaries and fibre optics could be interfaced to the microfabricated device for sample introduction and coupling light into and out of the device. Samples could be introduced into the fabricated device through a capillary using its inherent electroosmotic flow. The pinch mode was used to prevent sample diffusion into the separation channels during the injection period. High voltage was then switched to the separation channels for electrophoresis.

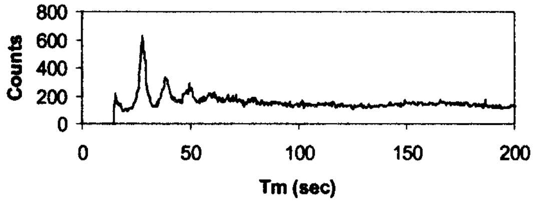

*The electropherogram of two NIR dye-labelled PCR primers using PMMA microchip.*

This device can be directly connected to a dynamic PCR system that uses fused silica capillaries as reactor. A mixture containing a PCR amplified fragment of λ-DNA was introduced to the micro-device after thermal cycling and analysed.

### Optically gated electrophoresis on a chip

*Julie A. Lapos and Andrew G. Ewing, Department of Chemistry, Pennsylvania State University, 152 Davey Laboratory, University Park, PA 16802*

Over the past decade, progress has been made in the application of micro and nanofabrication techniques to miniaturize analytical systems. Capillary electrophoresis (CE) in a chip has shown efficient separations comparable to those obtained in a conventional capillary. In addition, chip-based CE allows a high throughput of samples to be obtained because of the rapid time scale of the separation and the ability to fabricate arrays of separation channels onto a single chip.

An important parameter in chip-based CE is sample introduction. This research has focused on developing optically gated sample introduction on chips. In optically gated CE, fluorescently labelled sample is run continuously through the separation channel. A gating beam located near the entrance of the channel is used to continuously photobleach the sample. An injection is carried out by transiently blocking the beam to allow a small plug of unbleached sample through, which is then separated along the channel and detected with a probe beam.

Separation channels have been fabricated on Borofloat plates with an etched channel depth of 20 to 100 micrometers. Fluorescently labelled sample has been run continuously through the channel while using optical gating to introduce sample plugs. The narrow injection plugs, higher separation voltages, and reduced analysis time of this technique can be applied toward multiple sequential samples and continuous analyses which make this technique useful for biological and medical applications.

*Journal of Automated Methods & Management in Chemistry* ISSN 1463–9246 print/ISSN 1464–5068 online © 2001 Pittcon
http://www.tandf.co.uk/journals
DOI: 10.1080/14639240011003636 1

### An improved system for microchip electrophoretic separations of biomolecules with Raman spectroscopic detection

*Obianuju Inya-Agha and Michael D. Morris, Department of Chemistry, University of Michigan, Ann Arbor, MI 48109-1055*

We report on applications of an improved system for Raman-detected capillary electroseparations in the microchip format. Materials other than glass are investigated for signal enhancement and fluorescence background reduction. A redesigned chip holder allows for easy aligment and focusing of the system on the inverted microscope stage of a Raman microprobe.

Optimization of the laser delivery optics for planar chips improves the confocality of the detection system, and fluorescence background from the microchip itself is lower than in our first generation design. The redesigned optics also enable the use of lower laser power and faster signal acquisition than previously possible.

Typically, 1–3 Raman spectra per second are acquired during separation. The resulting sets of several hundred spectra are most conveniently interpreted after multivariate data reduction. Therefore, PCA-derived techniques are now used to extract component Raman spectra and to quantify analytes. We will illustrate performance of this Raman detection system using latest results from isotachophoretic and zone electrophoretic separation of ribonucleotides and other small ions of biochemical and biomedical interest.

### Real-time interactive self-modelling mixture analysis of ion mobility spectra

*Guoxiang Chen and Peter de B. Harrington, Center for Intelligent Chemical Instrumentation, Department of Chemistry & Biochemistry, Ohio University, Athens, OH 45701; e-mail: Guoxiang.Chen@ohio.edu*

Ion mobility spectrometry (IMS) furnishes low cost, sensitive, and portable sensors that afford a wide variety of potential applications. IMS sensors are fast, typically acquiring 20 to 30 spectra per second. Simple measurements may furnish up to several thousand spectra.

The IMS data often represent mixtures of several components. The simple-to-use interactive self-modelling mixture analysis (SIMPLISMA) has demonstrated to be an effective and efficient tool to resolve the spectral data of mixtures. Using SIMPLISMA in IMS, overlapping peaks may be resolved, and selectivity and sensitivity may be enhanced. However, the current SIMPLISMA methods have been based on offline processing prior to which a data storage process has been required.

In this work, an algorithm of near real-time SIMPLISMA that was developed by Rauch *et al.*[1] has been implemented as a real-time algorithm in LabVIEW. As a result, a real-time virtual instrument (VI) system has been developed to continuously acquire data from IMS and simultaneously extract spectra and concentration profiles by SIMPLISMA. The spectra and concentration profiles are displayed as the spectra are acquired on the user-friendly VI interface. The concentration profiles indicate changes of the individual component concentrations in the instrument response with respect to sample acquisition time and the spectra indicate the characteristic peaks of the components with respect to the ion mobility. This display allows subtle changes in the sensor's response to be easily visualized as the data are acquired.

[1] Rauch, P., Harrington, P. and Davis, D., *Chemom. Intell. Lab. Syst.*, 1997, **39**, 175–185.

### Chemometric techniques to improve the extraction of kinetic information from overlapped signals

*Ernst Bezemer and Sarah C. Rutan, Department of Chemistry, Virginia Commonwealth University, Richmond, VA 23284*

The determination of kinetic profiles of an evolving chemical system is used to obtain reaction rates in mechanistic studies. However researchers are not always fortunate that the changing component is observable without interference from the other species present. This research describes the chemometric tools to extract the kinetic profiles from multiple analyses with overlapping signals.

Time dependent data of poorly resolved chromatograms from HPLC-DAD as well as NMR data in which the species have nearly identical signals will be used to demonstrate the utility of the chemometric algorithms. The hydrolysis of one sulfonylurea herbicide (Ally) is chosen for this purpose. Reverse phase HPLC can not easily separate the hydrolysis products. The suspected products also have very similar $^{1}$H-NMR as compared to the parent compound. This makes the determination of kinetic profiles difficult using classical methods.

Evolving Factor Analysis (EFA) and Alternating Least Squares (ALS) are applied to these three-way data-sets in order to obtain the species dependent kinetic profiles. The choice in number of factors as well as the applied constraints such as non-negativity and trilinearity will be discussed.

### PC-based automation for preparative countercurrent chromatography

*F. E. Chou[1] and Y. Ito[2], [1]Pharma-Tech Research Corporation, Baltimore, MD 21212, USA and [2]Laboratory of Biophysical Chemistry, NHLBI, NIH, Bethesda, MD 20892, USA*

Liquid–liquid partition is a unique, efficient and cost-effective separation technique. Countercurrent distribution (CCD) based on liquid–liquid partition principle has been used widely and still been used today for purification of peptides up to ton quantities in the industrial settings lasting days to weeks.

The application of centrifugal force and specially designed coiled column enables one to perform countercurrent chromatography (CCC) for preparative separation at a laboratory scale (gram quantity) within hours instead of days. Due to slower flow rate (5 ml/min) for CCC operation comparing to that of HPLC, the improvement of CCC operation can be achieved through automation by interfacing with PC for monitoring operation and data acquisition. We use our developed DOS software to prove its feasibility. Due to widespread use of window software, we acquired a commercial software from Americhrom Inc. to evaluate the performance of our newly built prototype CCC (5000 ml) to large-scale preparative separation.

The engineering aspects for the fabrication of this prototype apparatus and preliminary results of automated separation will be presented and discussed.

### Ambient air monitoring of respirable particulate matter ($PM_{10}$) and total suspended particulate (TSP) during the RD180 rocket engine tests

*John Hughes and Kathy Lehr, Science Services Laboratory, John C. Stennis Space Center, MS 39529*

The Sciences Laboratory, located at Stennis Space Center and operated by GB Tech, was tasked by the Environmental Office at the NASA Marshall Space Flight Center (MSFC) to assess the environmental impact of air particulate matter as a result of RD 180 engine testing. The method used for monitoring ambient air included measuring particles smaller than or equal to 10 micron ($PM_{10}$), particles with diameters smaller than or equal to 2.5 microns ($PM_{2.5}$) and Total Suspended Particles (TSP) that have a diameter size of 25 to 50 microns. This particulate matter represents small 'dust like' particles of soot which are sufficiently small to disperse essentially like a permanent gas. Due to their small particle size, these particulates can be inhaled. The airborne concentrations are regulated and set by the EPA to prevent adverse effects associated with the inhalation of high levels of particulates.

Dedicated samplers were used to collect the correct particle size from the ambient air. The samplers also maintain a controlled flow rate to determine the volume of air sampled. Particles were collected on micro quartz or glass fibre filters that are equilibrated and weighed (tare) and after (gross) sampling to determine the weight (net mass) gain of the sample. The concentration of the particles captured from the air is then computed as the net mass collected by the volume of air sampled. Location of the samplers was based on plume modelling diagnostics and real-time atmospheric conditions of wind speed and direction.

This method provides a measurement of the mass concentration of airborne particulate matter for the determination of compliance with National Ambient Air Quality Standards.

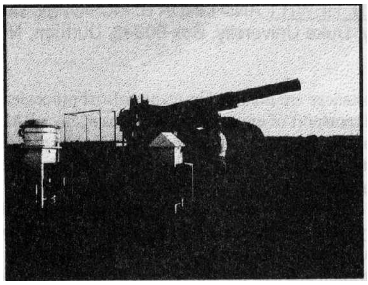

*Particulate samplers at collection site #2 at Redstone Arsenal.*

### Real-time, on-line characterization of particulate matter from combustion emission sources

*Paul Nam[1], Hongyu Shen[2], Shubhen Kapila[1,2], Philip Whitefield[2,4], Don Hagen[3,4] and Steve Chelli[5], [1]Center for Environmental Science & Technology, [2] Department of Chemistry, [3]Department of Physics, and [4]Cloud and Aerosols Science Lab, University of Missouri-Rolla, MO 65409, [5]Deposition Research Laboratory, Inc., St. Charles, MO 63301*

Monitoring of particle emission from combustion sources such as jet engines, automobile engines, power generators, etc., are necessary not only for their environmental impact but also for the optimization of their performance. The Mobile Aerosol Sampling System (MASS) developed by incorporating a differential mobility analyser (DMA), a multi-stage virtual impactor (MSVI), and a condensation nuclei counter (CNC) was integrated with mass spectrometers for on-line physical and chemical monitoring of particular matters (see the figure). An inductively coupled plasma ion source and a desorber/enrichment device were utilized as interfaces. This tandem arrangement permitted rapid determination of size distribution, relative abundance, and chemical composition of sub-micron size particles from combustion emission in real time. The system was used for characterization of sub-micron particles emitted from four-cycle and two-cycle automobile and small internal combustion engines. The system is being evaluated for on-line, real time determination of germanium and other metals and metalloids in fly ash from coal fired power plants. The use of the system permits rapid determination of elemental constituents of sub-micron particulates without laborious sample collection and sample preparation steps.

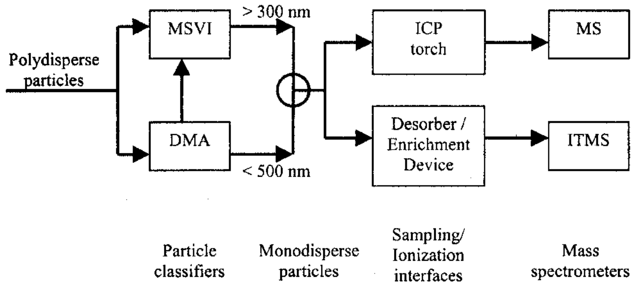

*Schematic representation of the particulate matter characterization and monitoring system.*

### Real-time detection of metals in individual airborne particles using mass spectrometry

*Philip J. Silva, Don-Yuan Liu, Christopher A. Noble and Kimberley A. Prather, Department of Chemistry, University of California, Riverside, CA 92521*

Traditional methods for determination of metals in atmospheric particles rely on the collection of particles on filters. Sample collection is followed by an off-line chemical analysis technique such as x-ray fluorescence or instrumental neutron activation analysis. This results in a time and composition averaged sample due to the sampling time required to obtain enough particles for analysis. The ability to perform rapid time measurements of metals in the atmosphere is limited in this way.

Recently, a number of techniques based on real-time single particle mass spectrometry have been developed that significantly increase the ability to detect rapid changes in particulate chemical composition. Aerosol time-of-flight mass spectrometry (ATOFMS) obtains chemical composition and particle size information. It can do this on rapid time scales (ten-minute resolution), thus providing particle size and chemical composition data with a fast time response.

Data acquisition with ATOFMS shows that on an individual particle basis, metals are present in different particle types with very different size distributions as opposed to the compositionally averaged data that one might obtain from traditional particle sampling. In addition, the long sampling time required with traditional sampling on filters (on the order of hours) can easily miss rapid changes in metal-containing particle concentrations.

Data from atmospheric particle sampling in Southern California will be shown to highlight the capability of ATOFMS for detection of metals in ambient particles, with a rapid time response. Focus will be placed on detection of trace metals such as vanadium, zinc, tin, lead, and barium. For each metal, observed size distributions and chemical correlations with other species will be discussed.

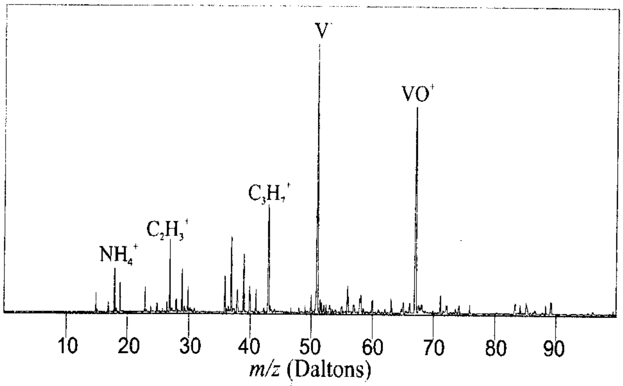

*Single particle mass spectrum of a 0.6 μm particle containing vanadium sampled from ambient Riverside air.*

**Automatic in-line calibration for IC: determination of anions in ultra pure water**

*Rachel E. Devine, Brinkmann Instruments, Inc., One Cantiague Road, Westbury, NY 11590*

The ion chromatographic determination of very low concentrations of anions using preconcentration techniques is standard practice in the semiconductor and power industry. Due to more sensitive ion chromatographic equipment, anions can now be determined in concentration ranges of single digit ng/L or parts per trillion. At such low levels accurate determinations are difficult due to contamination. Especially difficult is the preparation of standards for calibration.

With great care and skill, calibration curves from 1 ppb to 20 ppb with correlation coefficients of 0.9991 to 0.9999 can be obtained. Even using closed systems and automatic samplers, the errors due to contamination are great.

To overcome this problem we use a standard solution at the ppb level and perform dilutions in line. By flushing a 10 μL sample of the concentrated standard with 10 mL of water on to a concentrator column, we perform a 1000 fold dilution of the 1 ppb standard. To obtain a calibration curve, the standard is injected from one to ten times into the 10 mL of water during successive runs. The resulting calibration curves correspond to plots using the technique of standard additions. Thus the intercept of the calibration curve corresponds to the blank of the water used. This technique eliminates the blanks of the water used for setting up the standard solutions. Typical chromatograms using this technique, calibration curves and errors will be presented.

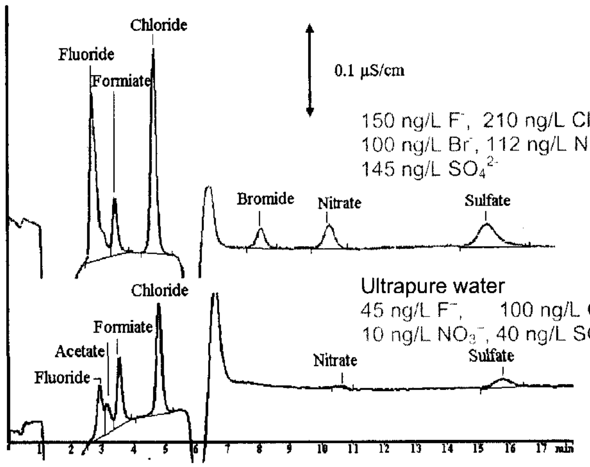

**LIMS, MES and ERP—an evolving laboratory IT landscape**

*Pat Tormey, Kevin Smith and Simon Wood, LabSystems, 1 St George's Court, Hanover Business Park, Altrincham, Cheshire WA14 5TP, UK*

The past few years has seen the general acceptance of large scale Enterprise Resource Planning Systems within organizations. The scope of these software products has grown such that they cover functionality from well outside of what was traditionally thought of as ERP functionality i.e. from financial systems and human resource management to document management and quality systems. The current trend would appear to be taking this approach a step further, to include MES (Manufacturing Execution Systems) and LIMS. While implementing a single system that seems to meet the requirements of the enterprise as a whole may seem appealing, there may be important issues involved within the laboratory that will not be accounted for if too high a level approach is taken.

An important area is the quality management of the data *within* the laboratory. Obviously the ERP keeps track of the quality of the manufactured product, but what about keeping track of standards that have been run? Control charts for instrument calibrations? Operator approval and SOPs?

Similarly, the need to interface instruments. Whilst a balance is relatively easy to interface, how sophisticated is the ERP's file parsing/mapping functionality, will it cope with a summary report from an ICP containing many samples and components?

Finally there is the question of the vendor's expertise of the business processes within the laboratory. These are very different from the processes that a typical ERP has to deal with.

The paper expands on these issues, and proposes some radical steps to ensure that the organization can implement an ERP, without compromising the timeliness and quality of laboratory data.

### Implementing LIMS—what does technology have to do with it?

*Nick Arnold and Simon Wood, LabSystems, 1 St George's Court, Hanover Business Park, Altrincham, Cheshire WA14 5TP, UK*

With LIMS, as with all other areas of information Technology and Information Systems, investments in new systems and projects can often be technology led, or if not technology led, technological issues assume a very high profile in the project. However, this type of technologically led approach to the purchase and implementation of systems is likely to have too narrow a focus and result in a system that fails to meet its objectives.

A technology led approach will often ignore organizational and people issues to the detriment of the project as a whole. However, it is often these issues that are, in fact, most important to the success of the project. This is because implementing a LIMS impacts people at all levels within the laboratory and can extend into many other areas of the enterprise as well.

A technology led approach will also tend to ignore business needs, and meeting defined business needs is fundamental to the success of any project. This paper defines in detail the issues surrounding technology, system implementation and project success. The role of the user community, the IS/IT department and the supplier are described as all three have a role to play in system implementation and project success. People issues including fear of new technology, resistance to change and internal politics are all factors that must be accounted for and managed during LIMS implementation, together with the need for buy in at all levels of the organization.

The point to remember is this; when implementing systems such as a LIMS that directly impact people on a day to day basis it is the people issues that are not only most important but also the most difficult to manage.

### Replacing the paper notebook

*Kevin Smith and Graham Taylor, LabSystems, 1 St George's Court, Hanover Business Park, Altrincham, Cheshire WA14 5TP, UK*

The objective of this research was to evaluate the use of Electronic systems as a replacement for the ubiquitous paper notebook.

For years scientists have relied on their paper notebooks as a medium for recording their work. Many have dreamed of a paperless solution, but the market for Electronic Lab Notebooks remains it its infancy. A small number of companies have developed in-house solutions, but commercial attempts to produce products have either been aimed at very niche markets, or have failed. It appears that the technology is now available to enable development of comprehensive Electronic Lab Notebooks, what remains is to identify the scope of the product. This paper gives an overview of the process we have undertaken to research the requirements, and then describes our view of the steps that need to be taken to make the ELN a commercially viable product.

To determine the requirements, we are consulting with industry groups (such as CENSA—the Collaborative Electronic Notebook Systems Association) as well as a number of end users from a wide range of industries. We have categorized the requirements into four broad areas.

(1) Knowledge Management.
(2) Legal and IP issues arising from the storage of records in electronic format.
(3) Compound document creation, including storage and manipulation of spectra and chemical structures.
(4) Ease and convenience of use.

We have then prioritized the requirements, and are in the process of addressing them in an evolutionary rather than revolutionary manner.

The paper will present our findings to date, and detail the follow up research that we intend to perform.

### Challenges of LIMS implementation in the millenium: experiences of a water utility lab.

*Ladun Odugbesan, BHC Company, 779 Main Street, Bridgeport, CT 06604*

BHC is the largest investor owned utility in New England and one of the ten largest in the US serving about half a million customers. The corporate laboratory is part of the Water Quality department and processes about 140 000 tests annually.

Prior to the purchase of the LIMS, the laboratory used a VAX operating system with an INGRESS database. The incentive to purchase a LIMS was initiated as a result of the Company's dynamic approach to Total Quality Management. Through benchmarking, BHC identified several Critical Success Factors (CSFs). The laboratory plays a key role in supporting one of the CSDs—Product Quality, and contributes to several performance measures that feed this CSF.

The LIMS project was developed by an Excellence-Through-Participation (ETP) team comprising of representatives from the Lab, Management, and Customer Service. Using the SMART principle, the ETP team developed project Performance Objectives.

This paper will look at the challenges faced during implementation of the LIMS in the laboratory. The continually changing operation of the laboratory to fulfil new regulatory challenges and the lessons learned from this experience.

### The role of suppliers project manager and project team in a LIMS implementation project

*Nicole Holliday, LabSystems, 1 St George's Court, Hanover Business Park, Altrincham, Cheshire WA14 5TP, UK*

The responsibility of a Project Manager is to implement LIMS Systems, including specific customer's requirements, within the constraints of time, costs, and resources. The Project Manager has a duty towards the customer, their own company and their team. They must deliver to the customer the required system at the agreed time and the system must meet the business needs. A high quality system implemented on time will be cost effective. A successful project will be morale boosting and will lead to a greater commitment from the team involved.

Careful planning is a vital prerequisite to success. A detailed project plan covering all aspects of the project must be agreed between the customer and the Project Manager at the start of the project, giving everyone involved a common understanding of the work ahead.

The user expectation must be managed all through the project. Too many requirements will lead to a legacy system, which will be difficult and costly to upgrade. 'The moving goalposts' syndrome, where requirements keep changing and increasing as the project progresses, must be avoided. User involvement is critical when prototypes are reviewed, as it is these users that will use the finished product.

Quality requirements must be precisely stated and agreed at the start of the project. It is to be expected that the software will be built so as to be compliant with those expressed requirements. It is essential to remember that poor quality results in the need to rework with all the cost, frustration and delay that this entails.

The project has a better chance of success if both the customer and the supplier work together. The Project Manager must communicate regularly with the customer, to ensure that the latter is aware of project progress. This is accomplished with emails, progress reports or telephone conferences, and makes for a more trusting and informed atmosphere.

Effective Project Management in Implementation is about ensuring that everything has been agreed, understood, documented and followed.

### Avoiding project failure in LIMS implementations

*Mark Fish and Jackie Taylor, LabSystems, 1 St George's Court, Hanover Business Park, Altrincham, Cheshire WA14 5TP, UK*

Implementing a LIMS in an organization is not the same as installing a new instrument in a laboratory. It is a much more complex task that can affect not just the laboratory or laboratory function but the enterprise as a whole. This makes the implementation of a LIMS a major IT project and major IT projects are notoriously prone to failure. Indeed from some of the reports that are available failure may almost be seen as the norm for IT projects.

However, implementing a LIMS does not need to be like this if the project is approached properly, and if the common reasons for IT project failure are understood and managed correctly. This paper explains in detail the common reasons for IT project failure including unrealistic time scales and expectations imposed on projects, changing requirements that are not managed correctly, lack of compromise and unwillingness to change. All of these factors are related to, and affected, by the constraints inherent in any project—these being the time available, functionality required (which can also be described as quality or fitness for purpose) and cost (or resources available). Practical examples of how the risk of project failure can be minimized will be provided including aspects of project management, the use of workshops for clarifying requirements and demonstrating solutions to those requirements, and the use of the tools to manage those requirements.

### Can a LIMS supplier ever meet your business needs?

*Simon Wood and Jackie Taylor, LabSystems, 1 St George's Court, Hanover Business Park, Altrincham, Cheshire WA14 5TP, UK*

The success of a LIMS implementation project should be judged on whether or not the business needs of the organization or enterprise have been met at the end of the project. However, there can be a tendency for clients implementing LIMS to make the supplier responsible for that success. This is not necessarily an issue provided that the supplier is actually allowed to make the project a success, although the chances of success are greatly increased when the client and supplier work as a true partnership or team.

Problems can arise when users do not fully understand what their business needs are and instead rely on strict adherence to the lists of functionality that typically form the contents of RFPs (Requests for Proposals) and ITTs (Invitations to Tender) as the measure of project success combined with on time and on budget delivery. This may give the client a system with the functionality that they think they need, but without knowing what the business needs are whether or not the system meets the needs cannot be judged.

The paper explains that this approach to measuring project success is flawed and proposes an alternative based more on meeting business needs. It also examines why the traditional approach to defining project success can, in fact, adversely impact the chances of project success. The difference beween business needs and functionality are also explored and an alternative approach to

LIMS implementation is proposed that helps to ensure that business needs are indeed met.

The answer to the question posed in the title of the paper is both No and Yes. No if the client does not understand what the business needs are and why they are important but yes if the business needs are known and understood and the client and supplier work together to meet them.

### True elemental speciation in liquid chromatography through particle beam glow discharge mass and optical spectrometries

*R. Kenneth Marcus, Department of Chemistry, Howard L. Hunter Chemical Laboratories, Clemson University, Clemson, SC 29634-1905*

Over the last decade, the topic of elemental speciation has been an integral component at meetings where atomic spectroscopy is the focus. The widely applied ICP-AES and MS approaches provide highly sensitive detection of components in a chromatograph containing various metals, but in the absence of extremely well characterized standards, cannot identify the actual chemical species eluting from an HPLC column. This low information content is coupled with comparatively high instrument costs and complexities versus the heart of the liquid phase speciation experiment, the chromatograph. Detectors for trace metal speciation via HPLC would ideally have the ability to identify not only the 'central' metal atom in an organic/inorganic complex, but the actual identity of the species as a whole, this is **true speciation**.

In this laboratory, we have coupled a particle beam LC-MS interface to a hollow cathode discharge source (PB-HC) to effect a new type of atomic emission and mass spectrometry detector for speciation studies employing HPLC separations. A diagrammatic representation of the basic system is shown below. In the case of AES, trace metals can be determined at the ppb level, with C and H (from ligands) being quantified at the single ppm level. Ratios of non-metal responses provide empirical formula data. For example, each of the natural amino acids can be identified by the ratios of the C/H/N components. Mass spectrometric sampling of HC plasma produces electron impact-type spectra for organic and organometallic compounds while free metal ions permit the use of isotopic patterns for identification and quantification through isotope dilution.

We describe here the analytical figures of merit for the PB-HC-AES/MS systems developed in this laboratory. The ability of the PB-HC couplings to be effectively employed to combined organic/inorganic sample types will be demonstrated through the analyses of dietary supplements and mixtures of organometallic compounds. While much work remains to be done to make this a truly viable approach, the overall importance of **true speciation** makes this a very important avenue of research.

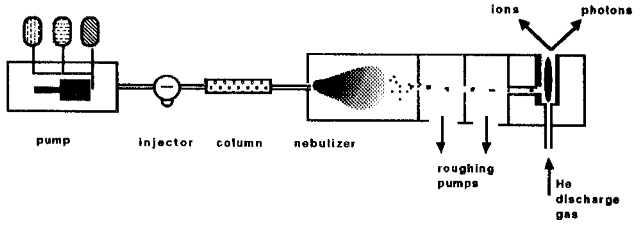

*Components of the particle beam–hollow cathode atomic emission/ mass spectrometry system.*

### High speed GC applied to Texas TPH samples

*Katrina Travis, Compuchem, 501 Madison Avenue, Cary, NC 27513; Karen E. LeBlanc and David W. Singer, Thermedics Detection, 220 Mill Road, Chelmsford, MA 01824*

Throughout the US, an enormous number of samples (both soil and water) are presently being submitted to analytical laboratories for TPH analysis. The TX TPH or EPA Methods 1005 and 1006 are the most commonly used methods for this analysis.

Temperature programming is presently considered an absolute necessity for this analysis. Conventional GC's have well documented maximum temperature programming rates. By using a simple accessory, the TX TPH method can be accelerated by as much as 15 times over the same analysis run on a conventional GC alone. The accessory that was used in the experiments that will be discussed, the EZ Flash, was installed in a Varian 3400 with a split/splitless injector and an FID.

The TX TPH method that was developed in this case was modified to include not only gasoline and diesel range organics, but mineral oil as well. Essentially, the method was modified to include a third range to the C6–C10 and C10–C28 ranges already specified in the above mentioned methods. This third range started with C28 and went up to and included C36. The data to be presented will demonstrate the ease with which this method was used to reduce the total cycle time for the analysis down to 3.5 minutes! Linearity and minimum detection limits along with data from blank extracts, spiked extracts, window defining standards, calibration standards, and real samples will be presented and discussed.

### Implementation of a new oil and grease automated extractor system for US EPA Method 1664

*Scott Frey, Christine Frazier and Bob Johnson, Horizon Technology, 8 Commerce Drive, Atkinson, NH 03811; e-mail: spe@horizontechinc.com*

In May 1999, Method 1664 for Oil and Grease was promulgated. The method had been under review for many years, which in hindsight, was a mixed blessing in disguise. The long review process resulted in many extensive public comment periods and suggestions, which resulted in the final method incorporating several analytical enhancements. One of the major enhancements was the allowance of Solid Phase Extraction as a directly equivalent extraction technique to the LLE procedure. SPE has been used for many of the drinking and wastewater methods, with proven success. The inclusion of SPE into Method 1664 indicates that SPE is clearly being recognized as a truly viable extraction technique for aqueous samples.

One of the major complaints with Method 1664, is that being a Performance Based Method, extensive QC requirements must be initially achieved to prove proficiency. Plus, this proficiency must be routinely demonstrated with each batch of samples. This places a considerable burden on the operators, as 'techniques' need to be perfected to consistently achieve acceptable recovery values. Due to the benefits of SPE, consistent recovery values of 96% and higher typically achieved.

The only downside to SPE, especially with typical wastewater samples, is trying to ensure the SPE disk does not plug. Coupling this problem with the desire to streamline the extraction process and provide as much automation as possible, a new automated Oil and Grease extractor system was developed. Utilizing SPE disks, this extractor system provides a level of consistency, which is difficult for operators to manually achieve over extended time periods. Plus, this system was designed to handle the dirty types of samples that typically have caused problems with earlier automated SPE procedures. This new system will be described in detail. Recovery data, sample throughput, and cost savings calculations will also be presented.

### Headspace analysis using Zymark laboratory robot

*Michael Markelov, Christopher Strauch, David Dechant and Olga Bershevits, ACS Labs, 5405 E. Schaaf Rd, Cleveland, OH 44131*

Very often the practice of headspace analysis demands sample preparation prior to equilibration of a headspace vial. This may include dissolution, addition of salts, adjustments of pH, spiking, derivatization, etc. No known headspace autosampler is capable of performing all of these manipulations automatically. The flexible automation available from the Zymark robot allows these varieties of manipulations to be carried out. It also allows repeated statistical analysis of samples in a cost-effective fashion. The robotic system presented can also be effectively used for headspace method development where degrees of dilution, amounts of spiking, etc. can be varied in a programmable fashion. This paper will present several examples from environmental, polymer, medical and food applications. One of them is the determination of parts per trillion levels of mercury in water.

This analysis is difficult to perform reliably using manual handling of samples and reagent especially by people with tooth fillings. It was found that some people's breath contains up to 10 ng/l mercury and will contaminate blanks and samples unpredictably. Automation at this level is a must.

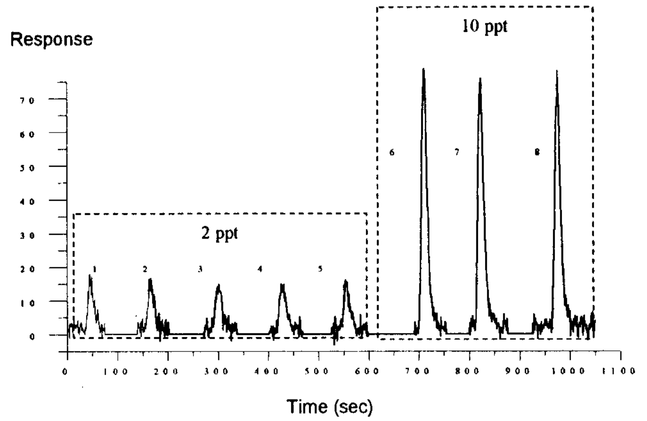

*Repeatability results for 2 and 10 ng/l solution (peaks 1–5 and 6–8, respectively) using a multi-pass cell in an RA-915 analyser. Sample volume is 10 ml.*

### On-line glucose monitoring using near-infrared spectroscopy

*Toshiyasu Tarumi and Gary W. Small, Center for Intelligent Chemical Instrumentation, Department of Chemistry & Biochemistry, Ohio University, Athens, OH 45701*

Near-infrared (NIR) spectroscopy has attracted significant interest as a potentially useful method for clinical analysis. Among the possible clinical applications, the non-invasive measurement of blood glucose levels by this method has been most extensively studied. NIR spectroscopy can also be applied to on-line glucose monitoring in clinical environments. Unlike conventional glucose assays using enzymes, NIR spectroscopy is non-destructive and reagent-free. It requires little or no sample preparation, resulting in a short analysis time. The method can easily be automated to perform continuous *ex vivo* blood glucose monitoring.

To realize a reliable on-line glucose monitoring method using NIR spectroscopy, the calibration model used to estimate the glucose concentrations must be optimal and robust. For example, the model must be able to correct for any baseline drift that occurs during the monitoring. Also, the variation of blood constituents other than glucose should not affect the glucose levels predicted by the model. In this study, the stability of the calibration model in the presence of these sources of variation is investigated using simulated biological sample matrixes. A flow cell is used to continuously monitor the concentration of glucose in the simulated matrixes. Multivariate calibration techniques are employed for the glucose analysis. The use of time-series analysis techniques and adaptive filtering methods will also be investigated for their utility in enhancing the stability of the calibration models.

### Improved process quality control via on-line analysis utilizing atomic absorption spectrometry

*Jophn Sanders and Thomas Preuss, Varian Australia Pty Ltd, 679 Springvalue Road, Mulgrave, Victoria 3170, Australia; Douglas Shrader, Varian, Inc., Optical Spectroscopy Instruments, 201 Hansen Court, Suite 108, Wood Dale, IL 60191*

Normally thought of as a laboratory based technique, Atomic Absorption spectrometry has been limited in its application to on-line monitoring and control situations in plant operation. However, there is a need for a simple 'press the button and go' operation in high volume applications where delays in presenting samples to a remote laboratory can cause unacceptable delays in control of plant processes.

The use of computer control and program integration allows the user to place an AA instrument on-line (or at-line), enabling plant operators to monitor trace and major metal concentrations almost instantaneously, for faster response in control situations. This operation can be as simple as Start and Stop buttons in a Microsoft® Excel spreadsheet, with Sequence and methods stored in the main AA software. Data is automatically transferred from the instrument software by OLE-2 transfer and processed in an Excel spreadsheet.

The application of this concept to real analyses will be illustrated with a cement analyser where control, operation and reporting of the SpectrAA hardware and software is run from Excel visual basic macro programs.

### On-line TOC monitoring of water purification processes

*Karen Clark, Michael Potts, and John Stillian, Anatel Corporation, 2200 Central Avenue Boulder, CO 80301*

Recent changes to drinking water regulations have increased the emphasis on using of Reverse Osmosis (RO) membranes for treatment of drinking water and wastewater. RO is a pressure driven process that virtually removes all ions and passes water. RO also has the potential for removal of all classes of pathogens, which is desirable for water treatment and allows for the possibility of water recycling. However, emphasizing the use of RO for microbial removal is a relatively new concept in the water industry, which has traditionally used these membranes for desalting. In addition, availability of accurate and sensitive real time methods for monitoring RO membrane performance and integrity has raised some questions. On-line conductivity measurement is one of the most commonly used techniques to monitor RO systems. Typically, a 1-2 log removal of ionic species can be measured by monitoring the influent and permeate conductivity of an RO system. However, there is a concern as to whether monitoring the influent and effluent conductivity can adequately warn of TOC or microbial breakthrough. The need for 'real time' measurements is becoming a necessity for water treatment plants as more facilities start using advanced water treatment processes. Real-time analytical data ensures immediate adjustments by operators to maintain optimum performance of the treatment process. On-line TOC analysis can be an invaluable tool in monitoring RO membrane performance and integrity. This paper discusses the operation of an on-line UV/persulfate TOC analyser and its application in monitoring RO performance. Data will be shown that demonstrates the ability of this analyser to detect more than a 2 log removal of TOC with RO membranes between influent and permeate streams. Thereby generating an additional log of sensitivity as compared to conductivity monitoring. This on-line analyser can measure TOC to less than 40 ppb providing low level detection and real time quantifiable results. This paper will also discuss the ability to correlate TOC removal with microbial rejection by RO membranes.

### The continuously variable volume reactor in flow injection analysis: theory, design, and application

*C. Chad Harrell, Elizabeth H. Medeiros and Stuart J. Chalk, Department of Natural Sciences, University of North Florida, Jacksonville, FL 32224*

Traditional flow injection analysis (FIA) experiments rely on a constant flow rate and a known reaction volume. This provides reproducible timing and dispersion of the sample and determines the maximum throughput of samples (based on the width of the peak). Analytical measurements are predominately based on peak height, and thus to improve peak height sensitivity a decrease in sample throughput is often necessary.

To avoid this problem we have developed the continuously variable volume reactor (CVVR). As the sample goes through the CVVR the volume can be changed independently of the volume segment of the injected bolus therefore changing the mixing volume dynamically. The upshot of this approach is that reaction/dispersion conditions can be optimized at both the peak maximum and the front and back of the peaks allow sensitivity to be maintained while 'squeezing' (decreasing the width) the peak and improving sample throughput.

In this paper we will present details of the design of the second generation CVVR and show the variations in peak shape (seen in the figure) that can be achieved using different chamber volume programs and mixing speeds. In the analysis of iron(II), we will detail the optimized chamber volume program, and demonstrate the reproducibility of the peaks obtained. Results of the analysis of iron(II) in environmental waters will be presented.

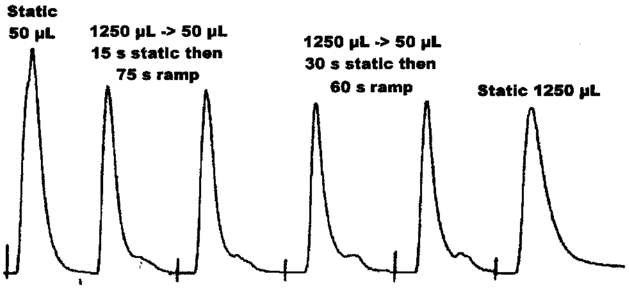

*Different peak shapes obtained from the CVVR.*

### Automated sample presentation systems for FT-NIR analysis of pharmaceuticals

*Cynthia Kradjel and Arnold J. Eilert, Bran+ Luebbe, Inc., 1025 Busch Parkway, Buffalo Grove, IL 60089*

The inherent speed and 'as is' analysis ability of near-infrared spectroscopy makes it an ideal technique for automated analysis. Numerous industries have taken advantage of this potential and are using near-infrared spectroscopy for on-line monitoring of industrial processes. However, automated near infrared analysis can also provide benefits in the laboratory. Several approaches to automated sample presentation have been developed for use in the pharmaceutical industry. Immersion probes or continuous flow systems are used for monitoring the progression of laboratory processes. Stop flow sample presentation is employed for the automated loading (via a peristaltic pump) of liquid samples into cuvettes that are designed for flow-through analysis. For solids, automated presentation of samples for diffuse reflectance analysis is achieved with pre-programmed positioning treys (for tablet analysis) and robotic replacement (for vials). Implementation of automation in the laboratory permits more samples to be analysed and frees the analyst for other activities. Various routine applications for which automated sample presentation schemes have been developed will be presented.

### In-line monitoring of emulsion polymerization and extrusion processes using fibre optic Raman spectroscopy

*M. S. Dhamdhere[1] and M. G. Hansen[2], [1]Department of Chemical Engineering, Univeristy of Tennessee, Knoxville, TN 37996 and [2]Department of Materials Science and Engineering, University of Tennessee, Knoxville, TN 37996*

In-line monitoring of chemical processes is important in order to develop precise and efficient control strategy for the process. Current off-line analysis techniques used for monitoring processes are time consuming and also lead to off-specification product, batch to batch variation in the finished product. In-line monitoring of processes yields almost instantaneous results. Current research is focused on the development of in-line monitoring tools for emulsion polymerization and extrusion processes using Raman spectroscopy as the analytical tool.

Emulsion polymerization is an industrially important process for the manufacture of polymer resins, paints and adhesives. In-line real time prediction of the residual monomer concentration during (co)polymerization of various acrylates is carried out in this research.

Results will also be presented for in-line real time monitoring of extrusion processes using polarized Raman spectroscopy. Prediction of rheological properties, e.g., melt Index (MI) of molten Ethylene Vinyl Acetate (EVA) and the correlation of vinyl acetate (VA) percentage in the copolymer is successfully carried out using polarized Raman spectra.

Chemometric techniques, such as Partial Least Squares (PLS), are used for developing the multivariate predictive models for prediction of residual monomer content in emulsion by polymerization and for predicting VA content, MI in extrusion process.

This methodology presents the new analytical techniques for better process monitoring resulting in considerable economic advantage.

### Chip-based integrated optics in laser spectroscopy

*Fred E. Lytle, Department of Chemistry, Purdue University, West Lafayette, IN 47907*

The successful resolution of many health issues depends upon diagnostic measurements. As the quantity of diagnostic tests increases, the drive for lower cost and reagent consumption will fuel a trend toward highly parallel, miniaturized instrumentation. This is best seen in electrophoretic separations, where published reports have demonstrated nearly one hundred, simultaneous DNA restriction fragment analyses using multiple channels etched onto a single quartz chip.

The goal of the described research is to improve performance of current, chip-based diagnostics by integrating detection optics onto the same chip as the electrophoresis instrumentation. Unfortunately, the millimetre dimensions of standard, integrated optical components are incompatible with the extremely close spacing of an etched array of electrophoretic channels. To circumvent this difficulty, an alternative approach to integrated optics has been developed which uses reflective waveguides, bends and beam splitters. Reflective waveguides represent a new paradigm in integrated optics as applied to chip-based instrumentation. Because they do not work on the principle of total internal reflection, it is possible to design and construct bends and splitters of micron dimensions. A comparison of normal and reflective waveguides will be presented, along with a brief discussion of the underlying optical theory.

In initial studies, integrated optical components were etched into a silicon master, which was then used to create plastic replicas. The optical performance of the replicated components will be discussed. Important parameters are waveguide insertion and propagation losses, bending losses, beam splitter losses, diffraction losses, and waveguide cross-talk. Alternative designs will be considered which have the potential to maximize optical throughput. The overall performance of the chip design will be determined by separating mixtures of diagnostic importance.

### Roles and responsibilities of the customer and supplier in LIMS validation

*John Dickson, LabSystems, 1 St George's Court, Hanover Business Park, Altrincham, Cheshire WA14 5TP, UK*

The validation of a Laboratory Information Management Systems (LIMS), or any other computer system, is a time consuming, costly and vital process both in the pharmaceutical industry and other industries subject to strict regulation.

The validation process must be treated as an integral part of system implementation. Indeed it must surround the implementation process: the validation approach must be approved before the project commences, and all validation documentation must be complete and approved before the implementation can be completed.

Validation is unavoidable in the pharmaceutical industry, but can also bring significant benefits, although costs are high. Validation effort as a percentage of implementation effort can vary widely within LIMS projects in the pharmaceutical industry, from below 10% to over 60%. There is an ongoing debate over what constitutes best practices.

For validation to be successful both the supplier and the client must understand their roles and responsibilities. The supplier has a vital role to play in the validation process but the client cannot pass all the responsibility for validation to them, or to any independent validation consultant.

Software validation entails structure ('white box') and functional ('black box') testing. In general, structural testing is necessary for unit and system tests. It requires access to source code, and detailed design documentation with a good understanding of both, and tends to be a vendor responsibility, with the quality confirmed by the cliant via a supplier audit. Functional testing is appropriate for user accepance testing, and tends to be a client responsibility.

While this distinction may seem clear, overlaps exist. All testing, must include stress and boundary condition testing. Thus, there are many similarities between testing required at unit, system and user acceptance test levels, and consequent opportunities to reduce validation effort. These opportunities can involve a collaborative test design approach, re-use of existing test material, and cross-reference to existing test evidence to avoid repeating identical tests. This approach can reduce validation cost without compromising its integrity.

With this co-operation, together with a clear understanding of responsibilities, excellent validation quality can be achieved without prohibitive cost.

### IQ, OQ, PQ and the pharmaceutical lab

*Kristi McKiney and John Lindahl, Scientific Software, Inc., 6612 Owens Drive, Pleasanton, CA 94588; e-mail: Kristi.McKiney@scisw.com*

The success of a pharmaceutical company in today's fast-paced drug discovery world depends on the ability of that company to streamline every step of its operation, including the important task of quality assurance. Because the use of chromatography data systems is crucial to the rapid discovery and development of new drugs, Installation Qualification (IQ), Operational Qualification (OQ), and Performance/Process Qualification (PQ) of these data systems (and the instruments attached to them) are difficult and important tasks. Such tasks can be greatly facilitated through tools provided by the data system itself and qualification services provided by the manufacturer. These tools must not only qualify installation of all software modules controlling the data system, but they must also provide a means for tracking day-to-day operations, down to providing answers to basic questions such as 'Does the instrument's actual flow rate match the set-point?'

In many companies, there is also a requirement for Design Qualification (DQ) which may require documentation of how the manufacturer controls the quality and functionality of the data system or instrument itself.

This paper will discuss the IQ, OQ, PQ processes and describe various tools and services available from Scientific Software, Inc. for the EZChrom *Elite* Client/Server data system.

### An organized approach to instrumental system validation

*Glen D. Wollenberg, Bruce Buchanan, and Eric Richmond, S21, 1.1.c, 216 Virginia Ave., Shenandoah, VA 22849*

The purchase of an instrumental system for use in a regulated industry is only the first step in the incorporation of the system into the owner's environment. System owners are often not prepared to qualify or validate a system for use in his or her environment. An integrated approach is needed requiring several disciplines which often must cross technical boundaries. Since the system owner is ultimately responsible for providing the documented evidence necessary to defend the system during an inspection, whether by regulatory personnel or internal auditors, an organized process for generating and providing this evidence is a paramount concern for the system owner.

Validation is a process, not an event, more of a marathon and not a sprint. The use of a System Lifecycle approach to instrumental validation, similar in concept to the Software Lifecycle approach of the Institute of Electrical and Electronics Engineers (IEEE), provides the system owner with the means for generating, accumulating, and organizing the documented evidence necessary to defend the system during an inspection. In essence, the system owner will be able to control the validation process and, as a result of the journey through this process, be able to provide documented evidence that addresses questions normally asked during an inspection or audit:

What is the purpose of the system?
How does the system address these requirements?
Who was responsible for creating the system and its components?
What are the system components and how are they controlled?
Was the system installed correctly?
Does the system perform its tasks as expected?

### Paperless GLP compliance in the chromatography laboratory

*James E. Schibler and Edward Long, Dionex Corporation, Sunnyvale, CA 94099-3603; e-mail: james_schibler@dionex.com and edward_long@dionex.com*

When the regulations of Good Laboratory Practices (GLP) were introduced in the 1970s, computers were very expensive and software was inflexible, so most calculations were done manually and results were stored in paper form. Since then, most laboratories have increased both the number of samples they analyse (through automation) and the amount of information they store for each sample (to protet themselves in the event of a GLP audit). As a result, the paper archives have become massive and unwieldy.

Over the past few decades, computers have become so powerful and inexpensive that nearly every chromatograph sold today is used with a chromatography data system. Although computerization has long promised to replace paper archiving with electronic storage that is compact and easy to manage, few regulated laboratories have actually achieved this goal.

The primary limitation has been traceability: users have not yet felt confident that their data systems will provide sufficiently clear, complete, and reliable documentation to pass a rigorous audit. They have been faced with inflexible security systems designed around the operating system rather than the chromatography workflow, incomplete validation of processes, and fragmented audit trails that make it difficult to show an auditor a clear, concise account of the laboratory work that was done.

This paper will discuss the data management, security, validation, audit trail, and electronic signature capabilities of a modern chromatography data system that was designed around the chromatography workflow, and explain how it facilitates GLP compliance in a paperless environment.

### A modular approach to instrument software control

*Frank Tontala, Shimadzu Software Development, 7102 Riverwood Drive, Columbia, MD 20146, and Dario Fiore and Steven Cubbedge, Shimadzu Scientific Instruments, 7102 Riverwood Drive, Columbia, MD 21046*

Traditionally, there have been two models used when designing software control drivers for analytical instruments. If the goal is to hide the details of the instrument command set, then proprietary software is written that encapsulates all aspects of the instrument interface and provides a monolithic one-vendor solution to the user. If the goal is to allow third party control of the instrument, then low-level ASCII commands are published that may be sent directly to the instrument via RS-232 or SCSI. Unfortunately, neither single solution allows both goals to be attained.

This paper discusses the use of a modular software approach to instrument control based on Microsoft ATL/COM (Component Object Model) programming. COM objects hide their core implementation, so that the user is insulated from the need to understand low-level communications protocols and commands. However, COM objects can also support a programming interface known as Automation. Automation provides access to instrument control by application level programmers using languages such as Visual Basic, as well as by system programming languages such as $C^{++}$.

Implementation of this concept will be described for components of the Shimadzu LC hardware family. COM objects were created that allow control of LC instrument components such as pumps, detectors and ovens. These objects can be accessed in a variety of ways to provide efficient integrated control. Examples of how the model was applied to the development of various applications will be discussed.

### Agilent Cerity networked data system for chemical QA/QC

*Jonathan A. Welsh, Agilent Technologies, Wilmington, DE 19808*

**Fully supporting routine tasks**
Perfect for routine analyses, the Agilent Cerity networked data system for chemical QA/QC fully supports the everyday tasks of laboratory professionals. This data system, based on Microsoft® Windows NT® 32-bit architecture, is part of the Cerity family of chromatography software. The Cerity family ends the one-size-fits-all approach to software design by offering targeted applications that automate most routine tasks for particular laboratory environments.

**Features of this data system**
*A sample-centric user interface.* One intuitively easy-to-navigate screen puts the sample at the centre of the analytical process: the operator picks the sample, the sample automatically determines the method, and the method automatically selects the GC. Anyone can operate the system without making a mistake.

*One screen for eight GCs.* You can control up to eight GCs from a single software screen—at maximum data acquisition rates for all eight GCs. You can set all GC parameters, view real-time results, and check instrument status for any of the eight GCs without having to start a new software session.

*Dynamic access to another 24 GCs.* Through the software's ConnectAdmin screen, you can store up to 32 GC network addresses and gain dynamic access to any eight without having to shut down the software. This lets you quickly and easily see what is happening on any networked GC not controlled by another computer.

*Productive LAN communications.* The software uses industry-standard local area network (LAN) communications. You can control and monitor laboratory instruments from any networked PC for the most productive use of your time. You can also send reports to your customers instantly using the World Wide Web.

*Database architecture for secure and easy information storage and retrieval.* To ensure data intregrity and system reliability, a relational database stores together everything that relates to an analysis. Nothing is ever overwritten.

*Control of various GCs.* The sample-centric data system lets you control the Agilent 6850 GC, 6890 Series GC, micro GC, or 5890 Series II GC.

*Language options for ease-of-use.* Because not everyone speaks and reads English fluently, the sample-centric software and on-line help come in different languages so operators can easily understand what they see and what they are supposed to do.

*Web reporting.* Using the software's Internet Explorer 5.0 viewer, you can present graphics and text in any compatible file format such as HTML, Java™, Word for Windows, Excel, PowerPoint, or ASCII test.

*Low cost per GC.* The sample-centric software is remarkably affordable. And it does not require a high-powered, high-end, high-cost PC. Furthermore, it does not need link boxes, terminal servers, or additional software to connect with, control, and acquire data from your GCs.

*Easy migration of methods and data.* If you are using Agilent's ChemStation, you can migrate your methods and data to the sample-centric Cerity QA/QC data system—for minimal disruption to laboratory operations.

### A new chromatography data system for quality control

*Wolfgang Winter, Agilent Technologies GmBH, Hewlett-Packard Str. 8, D-76337 Waldbronn, Germany, and Linda C. Doherty and Stephen J. Andrews, Agilent Technologies, Inc., 1601, California Ave., Palo Alto, CA 94304-1111*

Laboratory productivity can be negatively affected by administrative overhead tasks caused by non-optimal workflow support in the laboratory's data systems. Automation of workflow has become the key productivity bottleneck in analytical laboratories. New, dedicated networked data systems that model the way analysts work in their respective environment minimize this bottleneck. Customer-centred design technologies were used to develop laboratory-specific applications, abolishing the traditional 'one-size-fits-all' approach to software development. The first applications of the new software family focus on chemical and pharmaceutical QA/QC.

For chemical and petrochemical QC laboratories, poor workflow support typically results in lower analysis throughput because complicated sample entry screens slow down the operators. Sample-centric design and networking technology solve the problem. Sample entry is dramatically faster when the operator merely has to enter the sample name and the system performs the rest. Significant time is spent to check the status of current analyses on instruments distributed throughout the plant. Obtaining status information is easy and fast when instruments can be connected directly to the network. Operators require less training when the software user interfaces adapt themselves to the job role of the user.

Today, many GLP/GMP regulated laboratories use manual calculations that imply error prone manual data transcription into other software packages like spreadsheet programs. Validation of external programs and their interfaces to chromatography data systems is a major cost factor in these labs. This problem can be solved by incorporating a 'custom calculator' in the data analysis method of the chromatography data system, thus eliminating the need for external calculations. This approach handles the varying requirements for method specific calculations and statistical evaluations elegantly and minimizes the validation effort.

### An innovative automated micro scale SPE technology coupled with GC-MS for online analysis of water samples

*Yongtao B. Li, Ed J. George, Earl M. Hansen and Jerry J. Thoma, Environmental Health Laboratories, 110 S. Hill St., South Bend, IN 46617, and Werner Martin and Peter D. Smith, LEAP Technologies, 205 W. Main St., Carrboro, NC 27510*

SPE-GC-MS has been a well accepted technology in environmental analysis. However, manual and semi-automated SPE-GC-MS analysis of water requires time-consuming sample preparation, use of a large quantity of solvents, major investments in laboratory space fume hoods, evaporators and solvent disposal. Large sample volumes must be collected, preserved and transported at high costs. The end result is long turn-around times and high analytical costs.

In this presentation, we will describe an automated sample preparation system using 96 well micro scale SPE plates, which is coupled to a large volume injection (LVI) GC followed by MS analysis. The total system is a truly online analysis of semi-volatile organic chemicals (SOCs). This new SPE-LVI-GC-MS system is able to automatically perform sorbent conditioning, sample extraction, analyte elution, extract injection and on-line analysis of SOCs in variable volumes of water. The capability of this fully automated SPE-LVI-GC-MS will be demonstrated. Steps required to optimize the SPE-LVI-GC-MS procedures to achieve satisfactory sensitivity and resolution will be discussed. Secondly, the method accuracy, precision, and sensitivity will be presented and finally, the correlation between the present method and USEPA method 525.2 will be presented.

The Micro scale SPE-LVI-GC-MS technology provides several advantages over existing macro SPE-GC-MS methods:

(1) costs associated with sample transportation and preparation is significantly reduced;
(2) method sensitivity, accuracy, and precision is greatly improved;
(3) turn-around time of analytical results is reduced;
(4) analyst exposure to toxic solvents is significantly reduced or totally eliminated.

### On-line speciation of mercury in flue gas using amalgamation-atomic fluorescence spectrometry

*W. T. Corns, P. B. Stockwell and D. W. Bryce, P S Analytical Ltd, Arthur House, Crayfields Industrial Estate, Main Road, Orpington, Kent BR5 3HP, UK, and Dennis L. Laudal, Richard L. Schulz and Stan Miller, Energy & Environmental Research Center, University of North Dakota, PO Box 9018, Grand Forks, ND 58202-9018, USA*

Accurate measurement of mercury speciation in utility flue gas is necessary to model the fate and transportation of mercury in the atmosphere, and to evaluate the effectiveness of mercury control technologies. Impinger based methods such as EPA methods 29 and 101A have been successfully applied to determine total mercury. Modifications to those methods have allowed differential speciation for elementary and ionic forms of mercury, however the results to date have raised more questions than answers. There is clearly a need within the industry to continuously monitor mercury emissions and therefore a reliable approach using atomic fluorescence spectrometry was developed.

A dual-stage purge and trap system will be described based on amalgamation atomic fluorescence spectrometry at elevated temperatures. A sample interface to distinguish between elemental and total mercury has been developed thus enabling speciation of ionic mercury by difference. Results will be presented for both pilot and bench scale studies and the effect of various flue gas composites on the accuracy of the measurements will be discussed.

A fully automated system has been evaluated and results compared to other CEMs and to Impinger methods. The system performed consistently and mirrored the variations found in the standard methods.

### Experiences with a new multipoint, on-line, purge-and-trap autosampler to provide automated continuous monitoring of VOCS at a regional water utility

*Michael L. Duffy, OI Analytical, P.O. Box 9010, College Station, TX 77842-9010, Sandy Johnson and Ron McClintock, West Virginia American Water Company, 24th Street and Ohio River, Huntington, WV 25703, and Johnathan McSayles, ORSANCO, 5735 Kellog Ave., Cincinnati, OH 45228-1112*

The analysis of drinking water supplies for very low-level (ppb) concentrations of volatile organic compounds (VOCs) by purge-and-trap (P&T) GC and GC/MS has been performed for over 30 years. Historically, most of these samples have been manually collected in standard 40-ml VOA vials and subsequently analysed in the laboratory following specific USEPA methods such as 8021, 8260, 502.2, 601, 602, 624, and 524.2

This process provides good monitoring capabilities but results in more of a 'snap-shot' of the existing conditions only at the time the sample was taken. Any occurrence of a spill or unplanned 'event' that may result in a high VOC concentration is not detected until the next manual sample is collected. By automating the sampling on either a continuous or more frequent basis, any 'event' will be detected more quickly and permit a more immediate response to correct the situation. In addition to a more constant monitoring of a water supply (drinking, process, discharge water), an automated system can also provide more accurate data and a considerable costs saving by eliminating both the sampling errors and costs associated with manual sample collection.

This paper reviews the experiences of using a newly designed P&T on-line sampler at a regional water utility. The six-stream multipoint sampler was used to automatically monitor both the incoming source water from the Ohio River and the outgoing drinking water leaving the plant. The source water is monitored for any type of VOC contamination that may come from the river, such as spills or industrial discharges, etc. The outgoing drinking water leaving the plant is monitored, mainly for trihalomethane (THM) levels, to ensure continuous compliance with regulations.

### Field air sampling with SPME

*Jacek A. Koziel, Mingyu Jia, Abir Khaled, Japeth Noah and Janusz Pawliszyn, Department of Chemistry, University of Waterloo, Waterloo, Ontario N2L 3G1, Canada*

New sampling and analysis methods for total volatile organic compounds (TVOCs), several target VOCs, and formaldehyde in indoor air were developed and tested in several indoor air surveys. These methods were based on the use of commercially-available solid phase microextraction (SPME) technology and both portable and laboratory gas chromatography (GC). Indoor air surveys included both grab and time-weighted average (TWA) sampling and were completed at seven locations using SPME and conventional National Institute for Occupational Safety and Health (NIOSH) methods. Sampling locations included residential houses, rental appartments, office and industrial workplaces. Measured air concentrations for TVOCs, target VOCs and formaldehyde ranged from high parts-per-trillion to low parts-per-billion and correlated well with those estimated by the NIOSH methods. Solid phase microextraction proved to be more accurate, faster, more sensitive and cost-effective in field sampling applications. This paper will present methodology and field sampling results for all indoor air surveys. The authors will discuss advantages and challenges involved with SPME applications in indoor air surveys. This research should be of interest to research, industrial and regulatory agencies, as well as to general public concerned with indoor air quality.

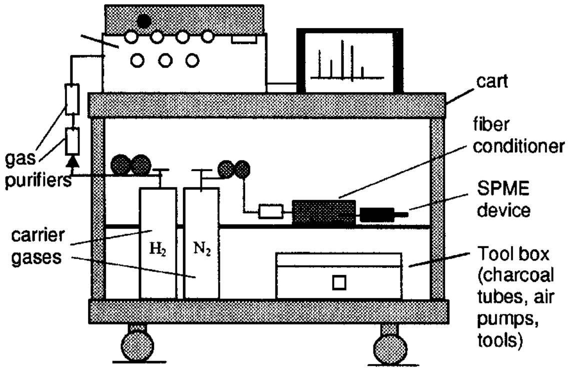

*Schematic of field air sampling system with SPME/GC.*

### Linking instruments to LIMS—a process of automation

*Phil Goddard, Contemporary Solutions Ltd, 13 Bolton Road East, Port Sunlight, Wirral CH62 4RU, UK*

Linking analytical instruments to LIMS is a process of automation. For effective solutions, the manual activities of a laboratory have to be matched *exactly*.

It is this emulation that represents most of the functionality of any generic linking solution. The more obvious issues of talking to the instrument and LIMS are trivial in comparison.

When instrument runs are set up and reported manually, the analyst takes account of a remarkable number of real-world issues as work passes through the laboratory. It is only when you try and automate these processes that the sheer scale of the job becomes apparent. Indeed, even the analysts are not consciously aware of just how much they do in this respect until it is coaxed out of them.

Clearly, it is important to have software to do the job that has the capabilities of operating in detail just as the human would have done. It is also generally critical to give the analyst overriding control over what is happening.

However, it is no use having particular abilities in software if they are not applied correctly on-site. Understanding the needs of the laboratory and its analysts and being able to configure the system so that it does what they want is paramount.

Finally, it is important to ensure that the implementation continues to match the ongoing needs of the laboratory—

even as the instruments, LIMS and analytical procedures evolve with time. Such changes also need to occur in a manner that brings minimum disruption to routine operation.

This paper gives detailed examples of instrument linking implementations from several key industries and application areas.

### On-line measurements of transformer gases in power plants

*John N. Driscoll and T. Bishop, HNU Systems, Inc., 160 Charlemont St, Newton, MA 02461*

A special GC has been developed for one-line *or* laboratory analysis of $O_2$ (oxygen), $H_2$ (hydrogen), CO (carbon monoxide), $CO_2$ (carbon dioxide), and the hydrocarbons $CH_4$ (methane), $C_2H_6$ (ethane), $C_2H_4$ (ethylene), $C_2H_2$ (acetylene). These gases may be evolved inside power transformers during operation, and the concentrations of these gases are monitored during operation, and the concentrations of these gases are monitored in order to assess the extent of deterioriation of transformer components or of the insulating oil.

The headspace samples over the transformer oil can be analysed on a continuous basis with the automated GC or a gas sample can be manually injected into the GC. The carbon oxides and hydrocarbon gases are separated on a chromatographic column. The carbon monoxide and dioxide are then converted to methane by passing the sample stream through a methanizer. Next, each of the gases is detected individually by a Flame Ionization Detector (FID). After passing through a separate column, the oxygen and hydrogen are detected by a Thermal Conductivity Detector (TCD).

Two columns, the methanizer, FID, and TCD are all incorporated conveniently in the GC. The method has been developed in such a way that the carbon oxides, hydrocarbons, and hydrogen are detected simultaneously. One injection of sample gas is divided so it passes through two separate columns, one leading to the FID, the other to the TCD. Oxygen is then detected by another injection of a gas which has been prepared specially for detection of oxygen alone. The GC operating parameters of oven temperature, carrier gas and column flow rate must be changed before acquiring oxygen data.

The retention times are as follows: FID: CO 1:18, CH4 1:45, CO2 2:55, $C_2H_2$ 4:45, $C_2H_4$ 5:00, $C_2H_6$ 6:45; TCD: $H_2$ 0:50. The detection limits for the FID are estimated to be between 0.3 ppm and 0.5 ppm. The $H_2$ detection limit is estimated at 100 ppm. The $O_2$ retention time is $1{:}18 \pm 0{:}03$. Estimated detection limit is 250 ppm. A typical chromatograph of transformer gases is given in the figure along with the conditions.

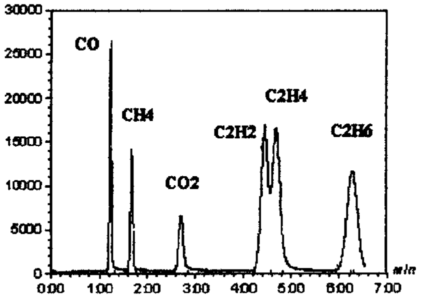

*Transformer gas analysis*

The reproducibility of the system (longterm precision and accuracy) and operating experience will be discussed in detail.

### Determination of mercury in the petrochemical industry

*W. T. Corns and P. B. Stockwell, P S Analytical Ltd, Arthur House, Crayfields Industrial Estate, Main Road, Orpington, Kent BR5 3HP, UK*

Natural gas and its liquid condensates are primary feedstocks for a variety of industrial processes. The presence of mercury in those samples is not just of environmental concern but also of economic importance. Heavy financial losses can be incurred by mercury induced corrosion of components used in the production, processing and transportation of natural gas condensates. Aluminium heat exchangers and downstream palladium hydrogenation catalysts are affected most dramatically.

The determination of mercury in natural gas will be reviewed. Off-line and on-line sampling devices will be described, based on amalgamation techniques with subsequent detection by atomic fluorescence spectrometry. Case studies will be presented showing the mass balance and control of mercury on gas processing plants.

More recently there has been a requirement for the determination of total mercury and speciation of liquid hydrocarbons. A novel procedure based on vaporization coupled to amalgamation at elemental temperatures will be presented. This approach eliminates the use of chemical additives and complicated digestion procedures which are normally required for total mercury. A system based on capillary GC-AFS will also be described for mercury speciation. Information gained from this technology provides a greater understanding of the distribution of mercury in petrochemical applications and has enabled the optimization of mercury removal strategies.

### Instrument development of portable atomic emission spectrometry using glow discharge and low pressure ICP: next generation

*Sang Chun Lee, *Seung-Cheol Lee, Jang-Soo Lee and Jin-Young Choi, Division of Chemistry and Chemical Engineering, Kyungnam University, Masan, 631-701, S. Korea; *Division of Biological Science, Kyungnam University.*

Glow discharge and low pressure inductively coupled plasma (ICP) was used to develop a portable atomic emission spectrometry (AES). See-through hollow cathode glow discharge (St-HCGD) cell for a portable AES was developed and the characteristics were studied. The excitation temperature at the St-HCGD cell was 6500–7000 K. Newly designed DC powered HCGD cell was examined for measuring the trace level of rare earth elements in solution. Most elements were tested for their detection limit with PMT and CCD detectors. The results were compared with the analytical data collected by ICP-AES. Operating and analysis programs of St-HCGD-AES were developed for a portable notebook computer. The St-HCGD-AES will be discussed its overall analytical performance and also will be compared with ICP-AES.

Meanwhile, low pressure-ICP was investigated its usefulness for a portable AES. We designed the low pressure-ICP torch and cell for 13.56 MHz and 27.12 MHz radio frequencies, and the analytical performance was investigated. We also tried to make a compact system with low pressure-ICP-AES but we still limited our effort for miniaturizing the system due to the bulky volume of a RF power supply.

Both plasma sources show good precision and accuracy for elemental analysis and we succeed somewhat miniaturizing the St-HCGD-AES and low pressure-ICP-AES. We will discuss the analytical performance and overall design of the portable AES.

### Continuous measuring of toxicity in waste water applications

*Meinolf Levermann and Dirk Köppenkastrop, Research and Development, STIP-ISCO GmbH, Siemenstraße 2, D-64823 Groß-Umstadt, Germany*

The following investigations deal with on-line measuring of toxicity in outlet streams of industrial plants. For that purpose an immobilized bacteria culture grows under the influence of sample and nutrients on small special shaped solids. Inside a reactor under constant temperature and constant oxygen concentration (measured by a dissolved oxygen probe) sufficient nutrients and biodegradable carbon compounds are added, so that the metabolism of the bio-mass according to the Michaelis–Menten kinetics is constant and independent from the nutrient concentration. Because of the special shaped solids it is guaranteed that the amount of bio-mass is constant.

Oxygen saturated tap water is added to the reactor to dilute the waste water sample. From the respiration behaviour of the bio-mass caused by sample composition and dilution factor toxic events in the waste water stream are detectable. Over and above the detection of toxic events the system is able to quantify the strength of toxicity of the sample. The dilution principal prevents the bio-mass from killing by toxicity.

With such a system examinations on the outlet stream of industrial plants were made. The detection and quantification of toxicity here is an extraordinary tool to protect the activated sludge in a following biological treatment plant to be sure the treatment process will work properly. With the knowledge of the strength of toxicity a toxic sample stream can be diverted to an additional basin. From here the toxic sample stream can gradually be added to the treatment process if the toxic event is over.

It was shown that the system works reliably and the information about the strength of toxicity is very suitable to protect biological treatment processes from being influenced by toxic events.

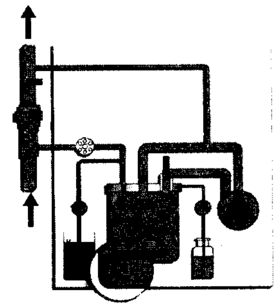

*Operation principle of the toxicity analyser.*

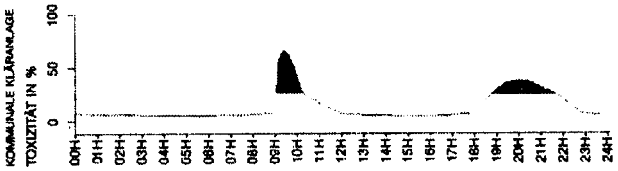

*Measuring trace with toxic events.*

### ChromSword Auto—a fully automatic method optimisation system for HPLC

*Reinhold Spatz, Volker Eckert and Sergej Galushko, Merck KGaA, SLP, 64271 Darmstadt, Germany*

Method development and Method optimisation in HPLC is expensive and time consuming. In most cases an expert is necessary who has many years of practical experience in HPLC.

The development target for this newly developed HPLC system was the fully automated method development without an expert using modules for Artificial Intelligence and intertechnology interactions. The proven theory based method development software ChromSword® takes control of reliable robust HPLC instrument modules.

For retention prediction and search for the optimum, mathematical procedures are used that take into consideration the theory of chromatography and the theory of optimization, based on Monte Carlo maps.

ChromSword® Auto can optimize HPLC methods based on the chemical struture of compounds to be separated, or based on practical experiments with standards of the analytes to be separated.

Theory, detailed working flow charts and typical experimental results from practical samples will be demonstrated. The results comprise automatic optimizations of the concentration of an organic modifier for isocratic and gradient reversed phase HPLC and temperature.

All results were achieved by fully unattended overnight and weekend work and without use of human expert knowledge.

Further perspectives in automated preparative separations and structure related predictions in combinatorial chemistry will be discussed.

### Automated tools for searching the world wide web and retrieving information

*C. S. Gilpin, Ohio University-Eastern Campus, Shannon Hall, 45425 National Road West, St. Clairsville, OH 43950 and D. J. Wagel and J. G. Solch, Brehm Research Laboratories, Wright State University, Dayton, OH 45435*

The focus of the current project as a practical review of the commercially available software programs and free web services that are available to scientists for monitoring the web and retrieving useful information. The free web based monitoring services are supported by advertising and provide notification of web page changes and some automated archiving. The software based internet agents are free or relatively inexpensive ($30 to $80) and include more sophisticated features such as personal spiders, filters, archiving functions and offline browsing.

This presentation will not pinpoint the best or worst tools, but will outline the different features and describe how they can be used to keep up with the constantly changing information and databases on the world wide web. The tools were tested in three different monitoring situations:

- Commercial sites for chromatographic column technology specifically related to environmental research
- Government sites for regulatory and funding information
- Topical sites for the humen genome projects

The ability of the tools to search and monitor several pages at a time on a regularly scheduled basis helps scientists keep up with the latest information without going from site to site to check for new products, regulations or other changes. By archiving web pages, the internet agents allow scientists to preserve and easily retrieve information that is important to their specific research. The following figure briefly summarizes the features of the tools investigated during this project.

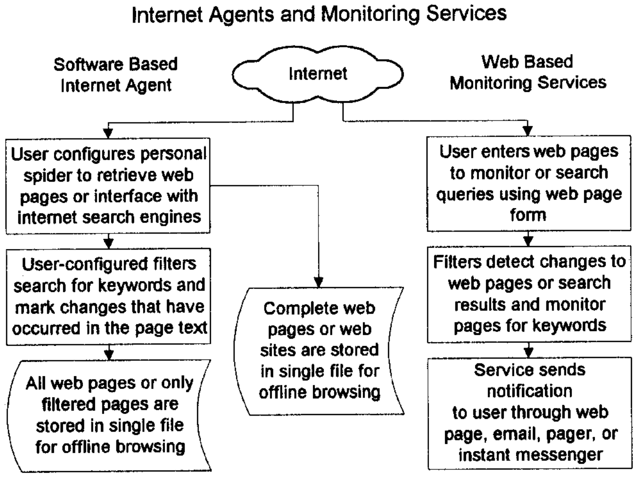

### On-line monitoring of water for aromatic hydrocarbons by UV derivative-spectroscopy

*Frank Vogt and Maurus Tacke, Fraunhofer-Institute of Physical Measurement Techniques, Heidenhofstr. 8, 79110 Freiburg, Germany*

The method 'Optical Derivative-Spectroscopy' is presented for supervision of legal emission limits of water for aromatic contamination. This technique makes use of a wavelength modulation that optically generates an approximation of the first and second derivative of the transmission. These derivatives are used additionally for evaluation in order to enhance spectral features for discrimination of the substances. This augmented spectroscopic technique is combined with chemometric algorithms. As application the selective measurement of aromatic solvents like benzene, toluene, chlorobenzene, ethylbenzene, and xylene isomers is demonstrated. From first investigations it can be estimated that the detection limits are well below 0.1 mg analyte per litre water by using 10 cm absorption pathlength and a few minutes measurement time. Selective monitoring is in contrast to the conventional 'Total Organic Carbon' method which determines only the total load of organic carbon in water. For ascertainment of the origin of environment pollution it is important, however, to know the composition of a contamination. For this purpose, a measurement in the UV spectral range (200–340 nm) is appropriate because

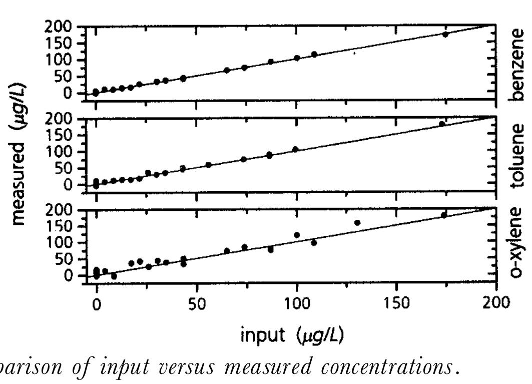

*Comparison of input versus measured concentrations.*

water has a high transparency in this wavelength range whereas IR-spectroscopic detection methods are hampered by the large absorption of water.

*Acknowledgement*: This work was financed by the German Environmental Foundation (ref. no. 6000/363) and the Max Buchner Research Foundation (ref. no. 2120). We also acknowledge Michael Jakusch and Boris Mizaikoff from the Institute of Analytical Chemistry, Vienna University of Technology, for advice concerning analytical chemistry and help in sample preparation.

